# Supplementary figures and images for: Comparative analyses of adsorbed circulating proteins in the PMMA and PES hemodiafilters in patients on predilution online hemodiafiltration
Source: PLoS One. 2024 Jul 19;19(7):e0299757. doi: 10.1371/journal.pone.0299757 (PMC11259279; doi:10.1371/journal.pone.0299757)

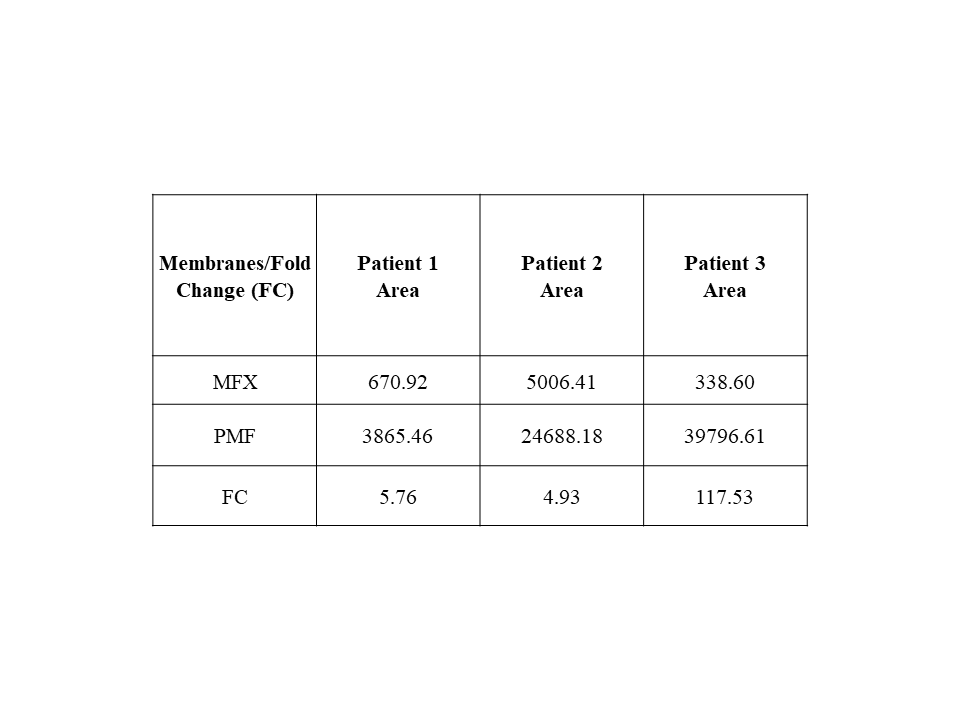

Supplement: S1 Table — Comparative detection of IL-6 is highly variable between the MFX and PMF membranes. The area value of each band of IL-6 on the MFX & PMF membrane was analyzed using ImageJ software. Here, FC = Fold change. (TIF) [file pone.0299757.s001.tif]

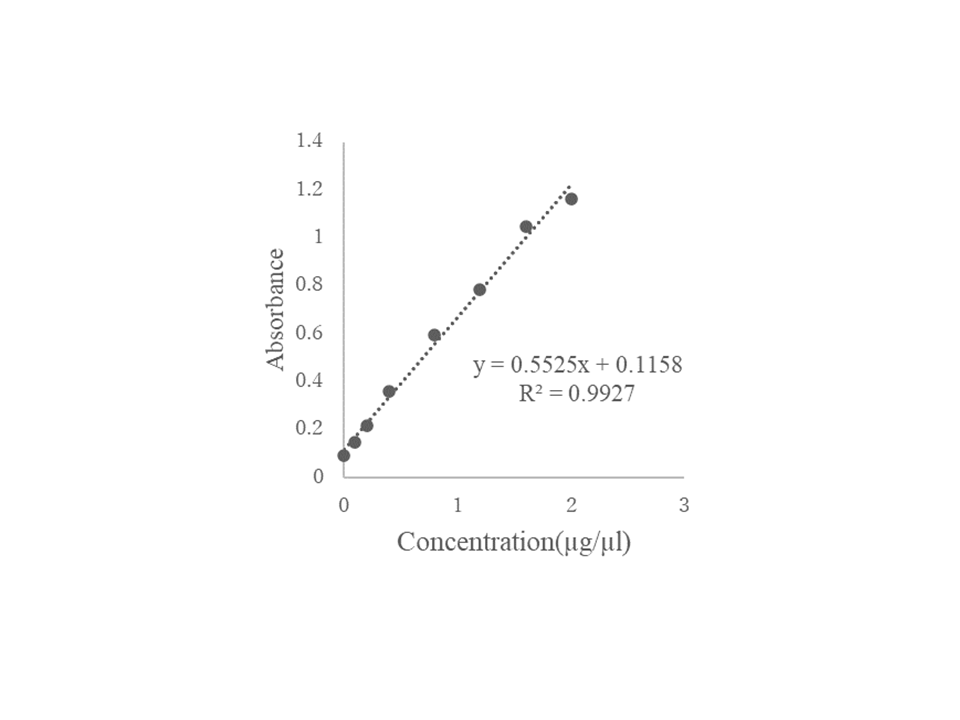

Supplement: S1 Fig — Calibration curve of BSA standard to determine the total protein concentration. (TIF) [file pone.0299757.s002.tif]

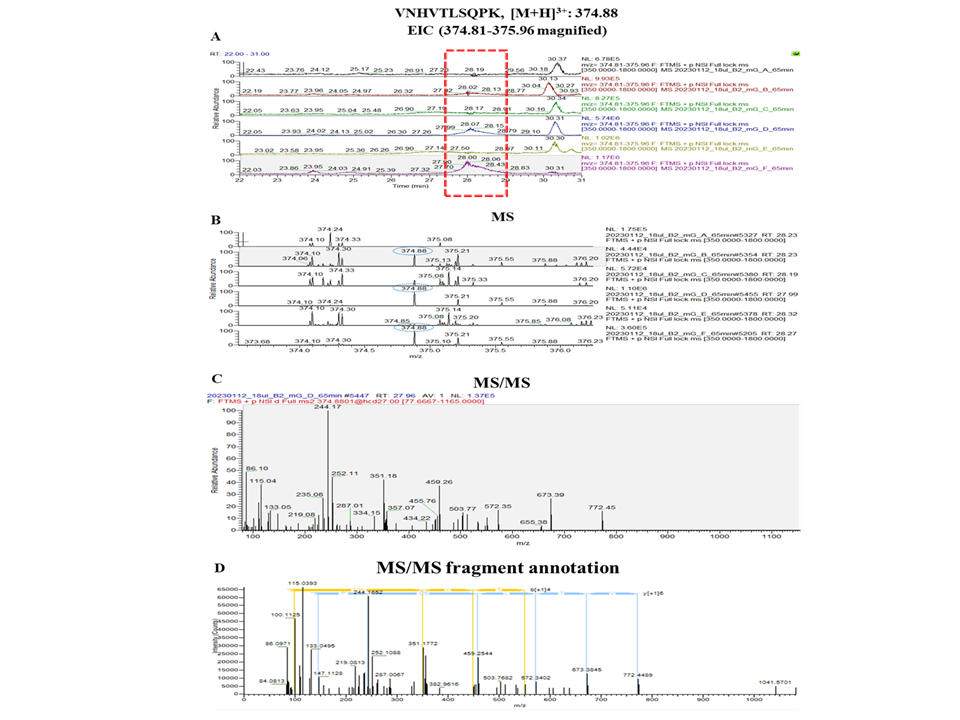

Supplement: S2 Fig — LC-MS/MS analysis of matched protein β2 MG. (A) Representative extract ion chromatogram (EIC) is shown hemodiafilter-bias ion m/z 374.81–375.96. Red squares show the ranges of retention time (RT) of targeted ions. (B) Mass spectra of target ions. (C) MS/MS spectra of target monoisotopic ions. (D) “VNHVTLSQPK” is identified as a hemodiafilter-specific peptide. (TIF) [file pone.0299757.s003.tif]

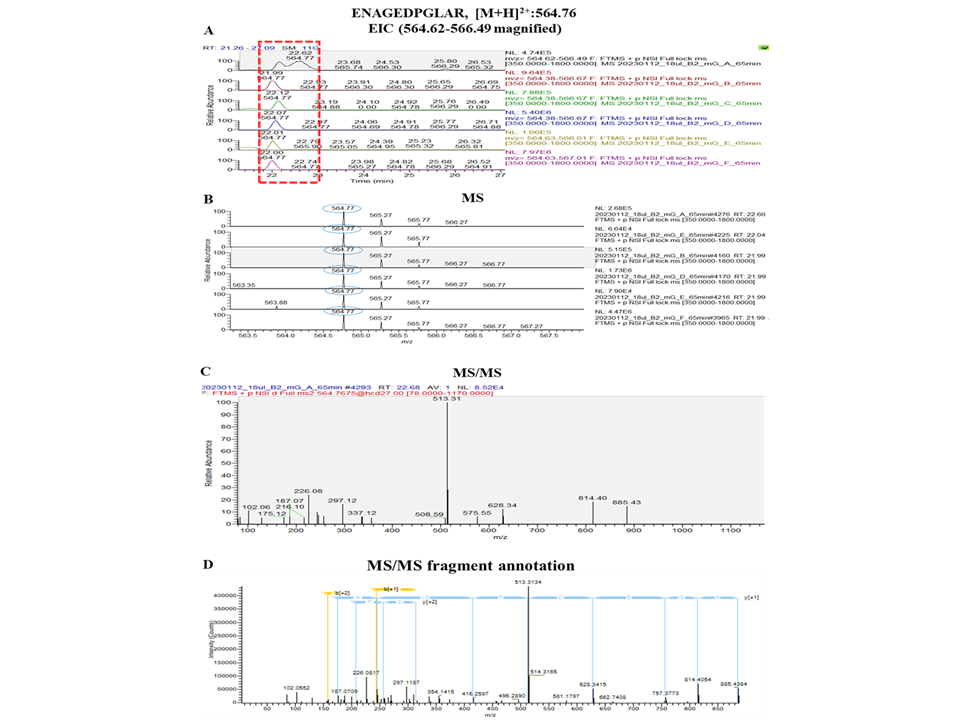

Supplement: S3 Fig — LC-MS/MS analysis of matched protein Dermcidin. (A) Representative extract ion chromatogram (EIC) is shown hemodiafilter-bias ion m/z 564.62–566.49. Red squares show the ranges of retention time (RT) of targeted ions. (B) Mass spectra of target ions. (C) MS/MS spectra of target monoisotopic ions. (D) “ENAGEDPGLAR” is identified as a hemodiafilter-specific peptide. (TIF) [file pone.0299757.s004.tif]

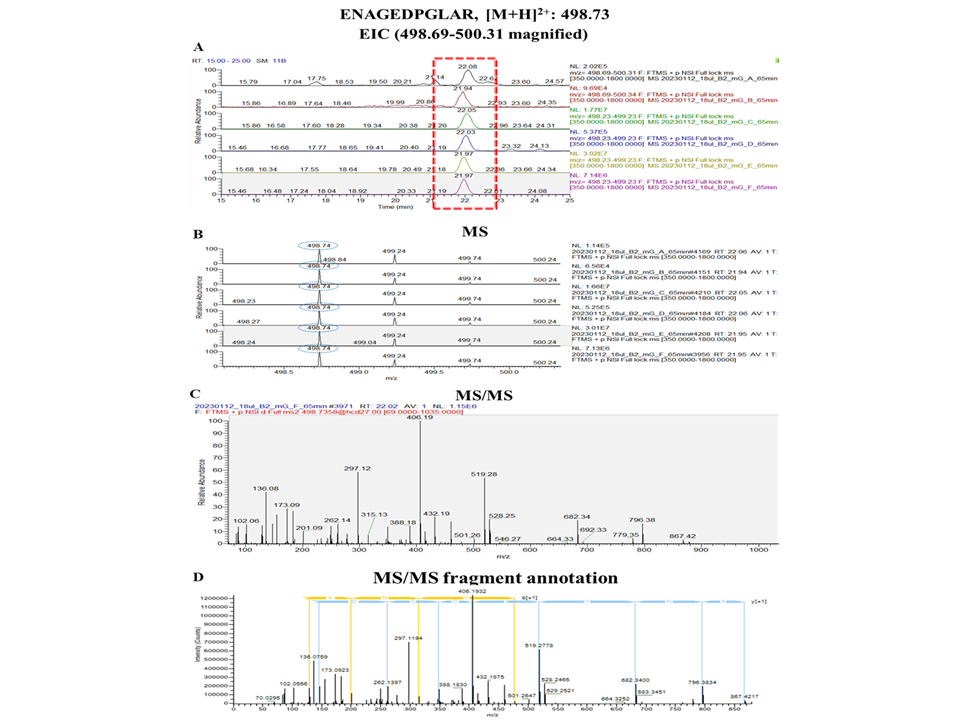

Supplement: S4 Fig — LC-MS/MS analysis of matched protein Serum amyloid A-1. (A) Representative extract ion chromatogram (EIC) is shown hemodiafilter-bias ion m/z 498.69–500.31. Red squares show the ranges of retention time (RT) of targeted ions. (B) Mass spectra of target ions. (C) MS/MS spectra of target monoisotopic ions. (D) “ENAGEDPGLAR” is identified as a hemodiafilter-specific peptide. (TIF) [file pone.0299757.s005.tif]

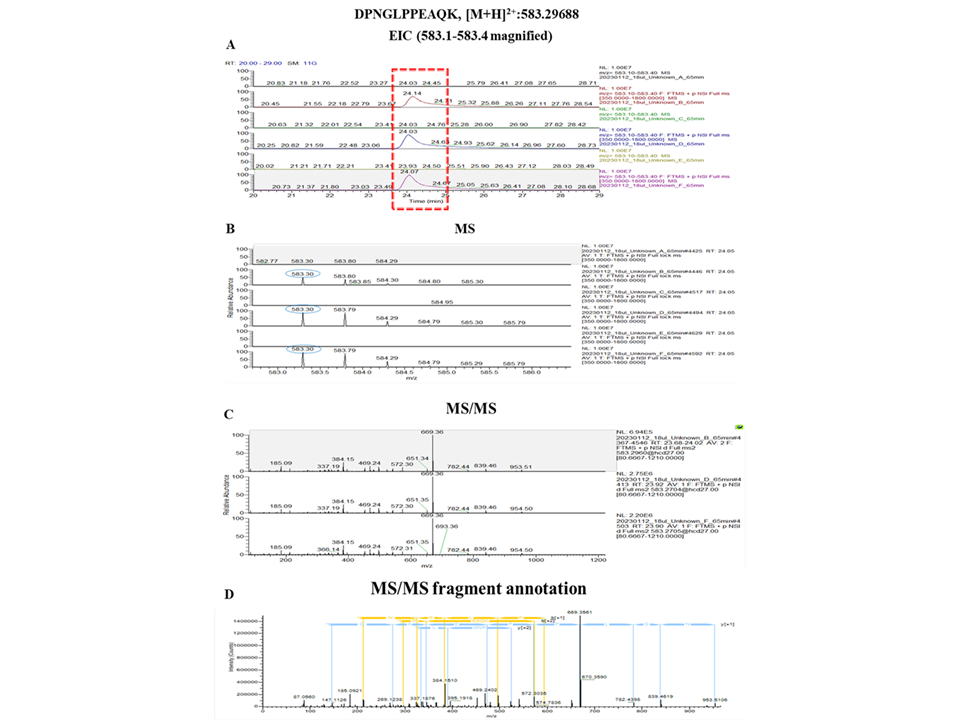

Supplement: S5 Fig — LC-MS/MS analysis of matched protein Retinol binding protein-4. (A) Representative extract ion chromatogram (EIC) is shown hemodiafilter-bias ion m/z 583.1–583.4. Red squares show the ranges of retention time (RT) of targeted ions. (B) Mass spectra of target ions. (C) MS/MS spectra of target monoisotopic ions. (D) “DPNGLPPEAQK” is identified as a hemodiafilter-specific peptide. (TIF) [file pone.0299757.s006.tif]

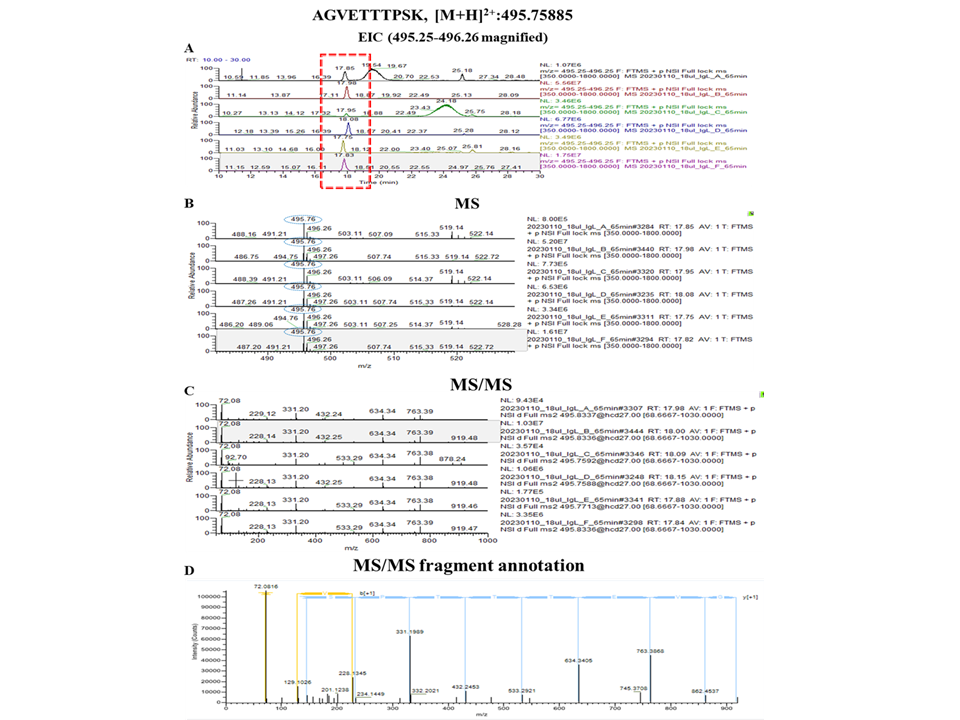

Supplement: S6 Fig — LC-MS/MS analysis of matched protein Immunoglobin lambda-1 light chain. (A) Representative extract ion chromatogram (EIC) is shown hemodiafilter-bias ion m/z 495.25–496.26. Red squares show the ranges of retention time (RT) of targeted ions. (B) Mass spectra of target ions. (C) MS/MS spectra of target monoisotopic ions. (D) “AGVETTTPSK” is identified as a hemodiafilter-specific peptide. (TIF) [file pone.0299757.s007.tif]

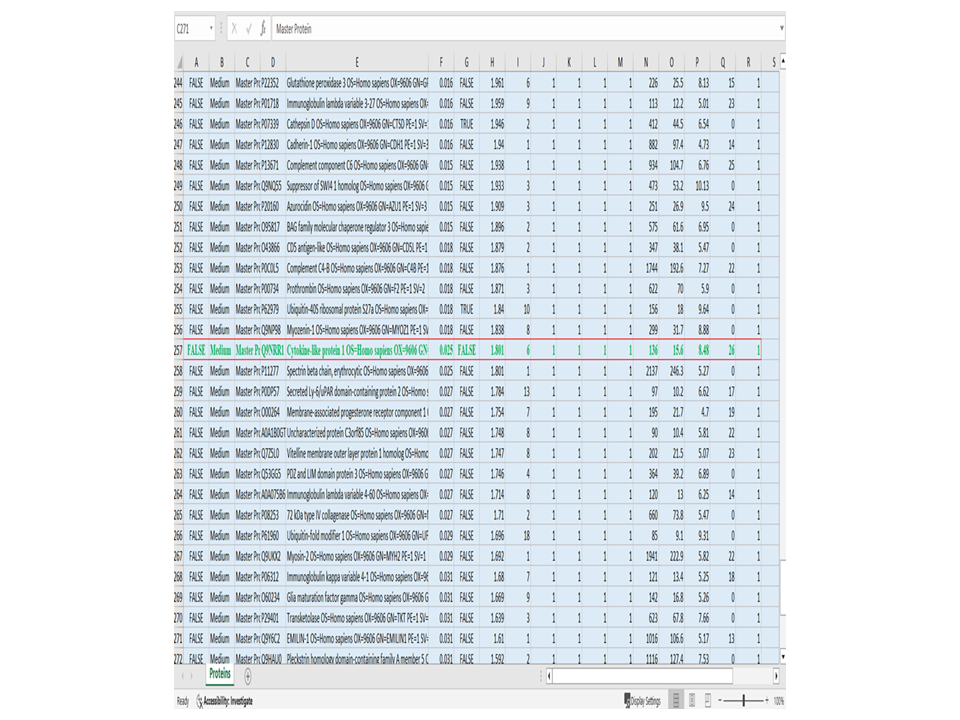

Supplement: S7 Fig — Matched protein Cytokine-like protein from the Excel sheet of Proteome Discover 2.2. (TIF) [file pone.0299757.s008.tif]
