# Supplementary material for: Comparative analyses of adsorbed circulating proteins in the PMMA and PES hemodiafilters in patients on predilution online hemodiafiltration
Source: PLoS One. 2024 Jul 19;19(7):e0299757. doi: 10.1371/journal.pone.0299757 (PMC11259279; doi:10.1371/journal.pone.0299757)
Supplement: S1 Raw images — SDS-PAGE of membrane extracts (Raw Fig 1) and Western blot analysis of adsorbed IL-6 on the dialyzer membranes (Raw Fig 2). (PDF) [file pone.0299757.s009.pdf]

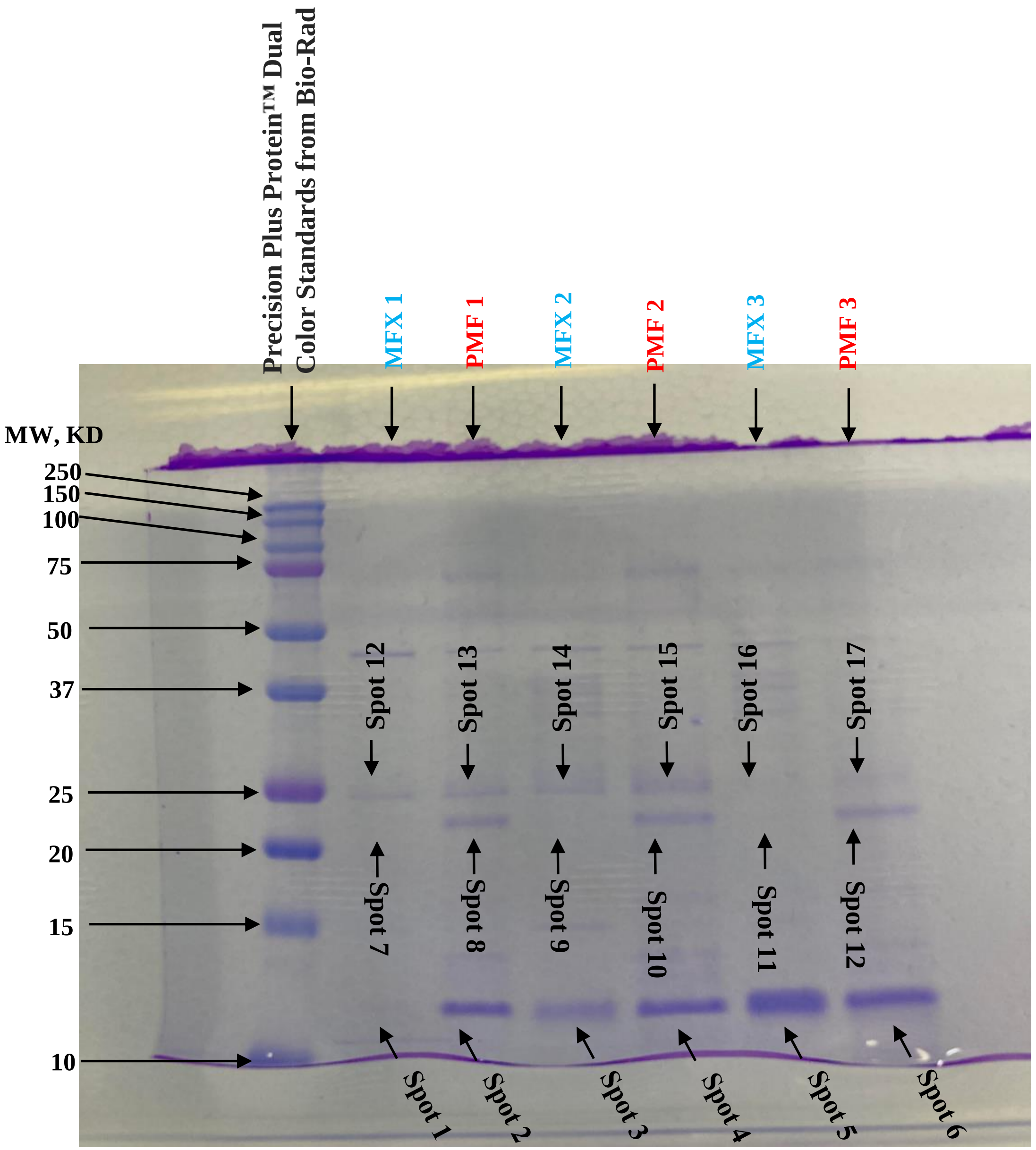

- The FUSION FX imaging system (Vilber Lourmat, Collégien, Seine-et-Marne, France) was used to take the image.
- Fig 1B is generated from this raw image.

Raw Fig 1- SDS-PAGE of PMMA (PMF) & PES (MFX) membrane extracts.

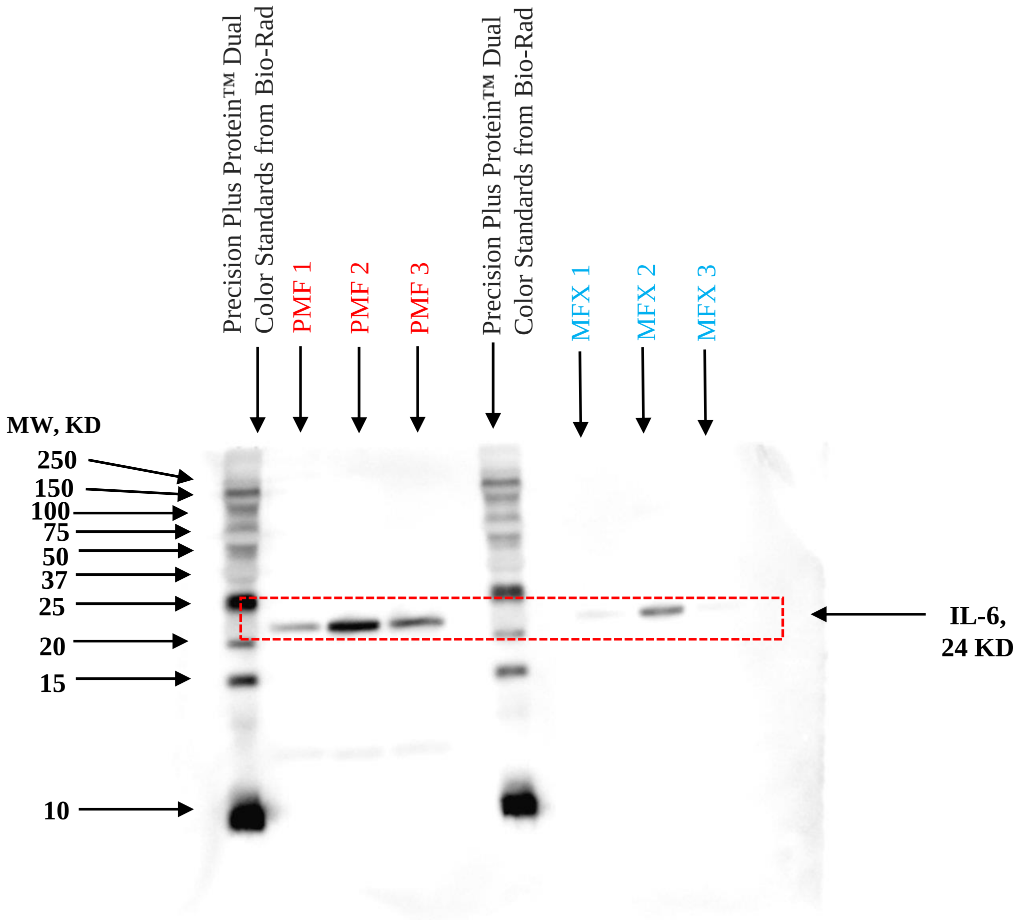

- The FUSION FX imaging system (Vilber Lourmat, Collégien, Seine-et-Marne, France) was used to take the image.
- Fig 3A is generated from this raw image.

Raw Fig 2- Western blot analysis of adsorbed IL-6 on the PMMA (PMF) & PES (MFX) dialyzer membrane.
